# Supplementary material for: Distribution of clones among hosts for the lizard malaria parasite Plasmodium mexicanum
Source: PeerJ. 2021 Nov 2;9:e12448. doi: 10.7717/peerj.12448 (PMC8570175; doi:10.7717/peerj.12448)
Supplement: Supplemental Information 1 — The first column lists the loci genotyped. The other four columns contain summary data for each site, given as number of distinct alleles (total number of alleles). For example, a locus with two infections, one with a single allele 147 and one with two alleles 147 and 153 would have two distinct alleles and three total alleles and be reported as 2 (3). [file peerj-09-12448-s001.docx]

Table S1: Number of alleles per locus at each of the sites sampled for testing the distribution of clones among hosts. The first column lists the loci genotyped. The other four columns contain summary data for each site, given as number of distinct alleles (total number of alleles). For example, a locus with two infections, one with a single allele 147 and one with two alleles 147 and 153 would have two distinct alleles and three total alleles and be reported as 2 (3).

Locus MLH GOR WT PC

Pmx306 9 (27) 9 (18) 9 (20) 7 (15)

Pmx732 8 (23) 6 (18) 6 (21) 5 (13)

Pmx747 8 (23) 9 (18) 7 (20) 7 (16)

Pmx839 10 (26) 7 (16) 8 (19) 5 (11)
