# Supplementary material for: Distribution of clones among hosts for the lizard malaria parasite Plasmodium mexicanum
Source: PeerJ. 2021 Nov 2;9:e12448. doi: 10.7717/peerj.12448 (PMC8570175; doi:10.7717/peerj.12448)
Supplement: Supplemental Information 2 — Results are reported as X2, P. [file peerj-09-12448-s002.docx]

Table S2: Goodness of fit results for individual sites for each distribution. Results are reported as X^2^, P.

Distribution GOR MLH PC WT

Poisson 13.2, **P<0.0014** 30.7, **P<0.0001** 51.98, **P<0.0001** 16.6, **P=0.0003**

NB 0.726, P = 0.696 3.48, P = 0.1758 11.91, **P = 0.003** 7.095, **P = 0.029**

ZI Poisson 0.085, P = 0.958 0.83, P = 0.659 5.96, P = 0.051 2.67, P = 0.260

ZINB 0.085, P = 0.958 0.83, P = 0.659 5.96, P = 0.051 2.69, P = 0.260
