# Supplementary material for: Distribution of clones among hosts for the lizard malaria parasite Plasmodium mexicanum
Source: PeerJ. 2021 Nov 2;9:e12448. doi: 10.7717/peerj.12448 (PMC8570175; doi:10.7717/peerj.12448)
Supplement: Supplemental Information 3 — Values are mu (m), theta (t), zero probability (z). The values of mu and theta for the ZINB are essentially the same as for the ZI Poisson, so only the theta estimate is reported for that model to save space in the table. [file peerj-09-12448-s003.docx]

Table S3: Fit values for individual sites for each model. Values are mu (m), theta (t), zero probability (z). The values of mu and theta for the ZINB are essentially the same as for the ZI Poisson, so only the theta estimate is reported for that model to save space in the table.

Model GOR MLH PC WT

Poisson 0.30 (m) 0.22 (m) 0.23 (m) 0.60 (m)

NB 0.30 (m), 0.17 (t) 0.22 (m), 0.17 (t) 0.23 (m), 0.15 (t) 0.60 (m), 0.57 (t)

ZI Poisson 1.48 (m), 0.80 (z) 1.18 (m), 0.81 (z) 1.31 (m), 0.82 (z) 1.50 (m), 0.60 (z)

ZINB 21,008 (t) 40,976 (t) 41,031 (t) 111,067 (t)

Simulation 1.18 (m) 1.10 (m) 0.91 (m) 1.25 (m)
