# Supplementary material for: Distribution of clones among hosts for the lizard malaria parasite Plasmodium mexicanum
Source: PeerJ. 2021 Nov 2;9:e12448. doi: 10.7717/peerj.12448 (PMC8570175; doi:10.7717/peerj.12448)
Supplement: Supplemental Information 4 [file peerj-09-12448-s004.docx]

Table S4: AIC and BIC for standard Poisson and NB models fit to data.

Model GOR MLH PC WT

Poisson 122 (AIC), 124 (BIC) 166 (AIC), 169 (BIC) 104 (AIC), 106 (BIC) 103 (AIC), 105 (BIC)

NB 103 (AIC), 106 (BIC) 145 (AIC), 149 (BIC) 90 (AIC), 92 (BIC) 99 (AIC), 100 (BIC)
