# Supplementary material for: Distribution of clones among hosts for the lizard malaria parasite Plasmodium mexicanum
Source: PeerJ. 2021 Nov 2;9:e12448. doi: 10.7717/peerj.12448 (PMC8570175; doi:10.7717/peerj.12448)
Supplement: Supplemental Information 5 — Vuong statistics and P values are reported raw as well as AIC or BIC corrected (see rows). Small P values indicate that the zero-inflated model is preferred. Large P values indicate that the models are indistinguishable. [file peerj-09-12448-s005.docx]

Table S5: Results for Vuong Test Comparing each zero inflated model to the traditional model. Vuong statistics and P values are reported raw as well as AIC and BIC corrected. Small P values indicate that the zero-inflated model is preferred. Large P values indicate that the models are indistinguishable.

Model GOR MLH PC WT

Poisson (raw) V = 2.21, **P = 0.014** V = 2.51, **P = 0.0060** V = 2.25, **P = 0.012** V = 2.86, **P = 0.002**

Poisson (AIC) V = 2.01, **P = 0.022** V = 2.32, **P = 0.010** V = 2.01, **P = 0.022** V = 2.41, **P = 0.008**

Poisson (BIC) V = 1.80, **P = 0.036** V = 2.04, **P = 0.020** V = 1.73, **P = 0.042** V = 2.01, **P = 0.022**

NB (raw) V = 1.11, P = 0.13 V = 1.72, **P = 0.042** V = 1.44, P = 0.0754 V = 2.90, **P = 0.002**

NB (AIC) V = 0.29, P = 0.38 V = 0.92, P = 0.18 V = 0.51, P = 0.305 V = 1.96, **P = 0.025**

NB (BIC) V = -0.66 P = 0.25 V = -0.23, P = 0.41 V = -0.58, P = 0.280 V = 1.11, P = 0.134
