# Supplementary material for: Distribution of clones among hosts for the lizard malaria parasite Plasmodium mexicanum
Source: PeerJ. 2021 Nov 2;9:e12448. doi: 10.7717/peerj.12448 (PMC8570175; doi:10.7717/peerj.12448)
Supplement: Supplemental Information 6 — Columns represent the minimum number of parasite clones (MC) per lizard host calculated as the maximum number of alleles at any of four microsatellite loci. For each site, I report: av.- the average number of simulated hosts containing a given number of clones over 1000 simulated samples of n… hosts; min. and max.- the minimum and maximum number of simulated hosts containing a given number of clones from any of the 1000 simulated samples; and obs.- the observed number of lizards containing a given number of clones. Observed values that fall outside the minimum and maximum simulated values are in bold. [file peerj-09-12448-s006.docx]

Table S6: Summary of observed MC vs. simulated MC for each site.

Columns represent the minimum number of parasite clones (MC) per lizard host calculated as the maximum number of alleles at any of four microsatellite loci. For each site, I report: av.- the average number of simulated hosts containing a given number of clones over 1000 simulated samples of *n_s_* hosts; min. and max.- the minimum and maximum number of simulated hosts containing a given number of clones from any of the 1000 simulated samples; and obs.- the observed number of lizards containing a given number of clones. Observed values that fall outside the minimum and maximum simulated values are in bold.

0 1 2 3 4 5 6 7 8 9 10

Site: GOR

# liz. (av.) 24 28 17 7 2 0 0 0 0 0 0

# liz. (min.) 12 15 7 1 0 0 0 0 0 0 0

# liz. (max.) 37 44 31 14 7 3 1 0 0 0 0

# liz. (obs.) **64** **5** **4** 2 1 0 0 0 0 0 0

Site: MLH

# liz. (av.) 43 48 27 10 2 0 0 0 0 0 0

# liz. (min.) 28 31 15 1 0 0 0 0 0 0 0

# liz. (max.) 60 67 43 21 9 3 1 0 0 0 0

# liz. (obs.) **113 7 9 0** 1 0 0 0 0 0 0

Site: PC

# liz. (av.) 31 29 14 4 1 0 0 0 0 0 0

# liz. (min.) 19 13 3 0 0 0 0 0 0 0 0

# liz. (max.) 48 45 28 11 4 2 0 0 0 0 0

# liz. (obs.) **68 4** 4 2 0 0 0 0 0 0 0

Site: WT

# liz. (av.) 13 16 11 4 1 0 0 0 0 0 0

# liz. (min.) 4 7 3 0 0 0 0 0 0 0 0

# liz. (max.) 23 26 19 13 5 2 1 0 0 0 0

# liz. (obs.) **31** **3** 9 2 0 0 0 0 0 0 0
